# Association between maternal social deprivation and prenatal care utilization: the PreCARE cohort study

**DOI:** 10.1186/s12884-017-1310-z

**Published:** 2017-05-16

**Authors:** Clémentine Gonthier, Candice Estellat, Catherine Deneux-Tharaux, Béatrice Blondel, Toni Alfaiate, Thomas Schmitz, Jean-François Oury, Laurent Mandelbrot, Dominique Luton, Philippe Ravaud, Elie Azria

**Affiliations:** 1 UMR1153 – Obstetrical, Perinatal and Pediatric Epidemiology (EPOPé research team), DHU Risks in Pregnancy, Paris Descartes University - INSERM, 53 Avenue de l’Observatoire, 75014 Paris, France; 2 Department of Obstetrics and Gynecology, Beaujon-Bichat Hospital, DHU Risks in Pregnancy, APHP, Paris Diderot University, 46 Rue Henri Huchard, 75018 Paris, France; 3 Epidemiology and clinical research Department, URC Paris-Nord, APHP, 46 Rue Henri Huchard, 75018 Paris, France; 4 CIC 1425-EC, UMR 1123, INSERM, Paris, France; 5 Department of Obstetrics and Gynecology, Robert Debré Hospital, AP-HP, Paris Diderot University, 48, boulevard Sérurier, 75019 Paris, France; 6 Department of Obstetrics and Gynecology, Louis Mourier Hospital, DHU Risks in Pregnancy, AP-HP, Paris Diderot University, 178 Rue des Renouillers, 92700 Colombes, France; 7 UMR676, Paris Diderot University - INSERM, Paris, France; 8 UMR1153 - Méthodes de l’évaluation thérapeutique des maladies chroniques (METHOS research team), INSERM, 1 Place du Parvis de Notre-Dame, 75004 Paris, France; 9 Department of Obstetrics and Gynecology, Groupe Hospitalier Paris Saint Joseph, DHU Risks in Pregnancy, Paris Descartes University, Paris, France

**Keywords:** Prenatal care utilization, Social deprivation, High-risk pregnancy, Social inequalities in health

## Abstract

**Background:**

Maternal social deprivation is associated with an increased risk of adverse maternal and perinatal outcomes. Inadequate prenatal care utilization (PCU) is likely to be an important intermediate factor. The health care system in France provides essential health services to all pregnant women irrespective of their socioeconomic status. Our aim was to assess the association between maternal social deprivation and PCU.

**Methods:**

The analysis was performed in the database of the multicenter prospective PreCARE cohort study. The population source consisted in all parturient women registered for delivery in 4 university hospital maternity units, Paris, France, from October 2010 to November 2011 (*N* = 10,419). This analysis selected women with singleton pregnancies that ended after 22 weeks of gestation (*N* = 9770). The associations between maternal deprivation (four variables first considered separately and then combined as a social deprivation index: social isolation, poor or insecure housing conditions, no work-related household income, and absence of standard health insurance) and inadequate PCU were tested through multivariate logistic regressions also adjusted for immigration characteristics and education level.

**Results:**

Attendance at prenatal care was poor for 23.3% of the study population. Crude relative risks and confidence intervals for inadequate PCU were 1.6 [1.5–1.8], 2.3 [2.1–2.6], and 3.1 [2.8–3.4], for women with a deprivation index of 1, 2, and 3, respectively, compared to women with deprivation index of 0. Each of the four deprivation variables was significantly associated with an increased risk of inadequate PCU. Because of the interaction observed between inadequate PCU and mother’s country of birth, we stratified for the latter before the multivariate analysis. After adjustment for the potential confounders, this social gradient remained for women born in France and North Africa. The prevalence of inadequate PCU among women born in sub-Saharan Africa was 34.7%; the social gradient in this group was attenuated and no longer significant. Other factors independently associated with inadequate PCU were maternal age, recent immigration, and unplanned or unwanted pregnancy.

**Conclusion:**

Social deprivation is independently associated with an increased risk of inadequate PCU. Recognition of risk factors is an important step in identifying barriers to PCU and developing measures to overcome them.

**Electronic supplementary material:**

The online version of this article (doi:10.1186/s12884-017-1310-z) contains supplementary material, which is available to authorized users.

## Background

Social deprivation increased in most OECD (Organisation for Economic Co-operation and Development) countries as a consequence of the economic crisis of 2007–08. More deprived populations have a higher risk of adverse maternal and perinatal outcomes [1, 2], such as non-chromosomal congenital anomalies [3], preterm birth or small-for-gestational-age status [4]. The mechanisms explaining these associations remain unclear, and many intermediate factors may be involved. Adequate prenatal care, assessed both by its timing and content, is believed to be an important factor in reducing maternal and perinatal risk. Inadequate prenatal care utilization (PCU) might thus be one intermediate factor. Because inadequate PCU is potentially modifiable through targeted interventions, it is of particular interest.

Although the number of prenatal visits can probably be reduced for women with low-risk pregnancies without consequences to maternal or perinatal health [5], some basic components of prenatal care can have a significant effect on the health of mothers and newborns [6]. Conversely, inadequate PCU is associated with higher perinatal morbidity and mortality [7, 8], especially among socially deprived women [9]. In addition, the effectiveness of special prenatal care programs adapted to the social and cultural specificity of women in deprived situations has been demonstrated [10–12].

Earlier studies of the association between socioeconomic condition and PCU have shown that women with social vulnerabilities start prenatal care later [13] and have fewer prenatal visits than other women [14, 15]. Numerous studies [13, 16–19] many conducted in United States of America [13, 16, 17], have identified social factors as barriers to PCU. Only a few, however, have described the association between maternal social deprivation and PCU. Moreover, most have reduced this multidimensional social condition to proxies such as ethnicity or insurance coverage, whereas we believe it needs to be considered more extensively, including several aspects related to social isolation, housing, income, and health insurance.

France, where institutions and policies prevent the imposition of basic economic barriers to prenatal care, through universal health care insurance or state medical assistance, is an interesting model for studying the role of individual factors in the utilization of prenatal care [20]. To our knowledge, no prospective study has ever addressed PCU specifically according to maternal social deprivation in France. Understanding the mechanisms that underlie the association between maternal social deprivation and PCU is essential to be able to propose concrete interventions to optimize PCU for women in deprived situations and reduce their risk of adverse pregnancy outcomes.

Our objective was to study the association between maternal social deprivation and prenatal care utilization. To achieve this goal, we conducted a secondary analysis of the French multicenter PreCARE cohort study.

## Methods

The study was approved by the regional ethics review board (CPP-Ile-de-France III). Each patient included in the cohort provided oral informed consent, in compliance with French law.

The PreCARE multicenter cohort study was designed to study the impact of maternal social deprivation on pregnancy and neonatal outcomes. It recruited all volunteers women registered to give birth at one of four maternity units in university hospitals in the Paris North area (France) from October 2010 through November 2011. During the study period, 10,779 women gave birth in the participating centers and 10,419 women and their newborns were included in the PreCARE cohort study (96.7%). For this analysis of the association between maternal social deprivation and PCU, we restricted the study population to women with singleton pregnancies delivered after 21 completed weeks of gestation (*n* = 9770).

### Data collection

A self-administered questionnaire completed at cohort enrollment collected data about social conditions (Additional file 1). In case of missing data, information was retrieved from a second questionnaire self-administered during the postpartum hospitalization (Additional file 2). Questionnaires were available in the four most common languages of these hospitals’ patients. A non-medical research assistant and interpreters were available if women needed explanations or had difficulty reading or completing the questionnaires. Data about PCU were collected in the postpartum questionnaires and from information in women’s medical files, extracted by the research assistants. The obstetricians or midwives who cared for the women collected their medical characteristics and information about their pregnancies.

### Definitions of maternal social deprivation

Maternal social deprivation was characterized by four binary variables measuring four dimensions of deprivation at the beginning of the pregnancy: 1) social isolation (the woman did not expect support from a friend or family member for activities of daily life after the baby’s birth); 2) insecure or unstable housing situation (the woman did not have her own housing, that is, in a residence owned or rented by her or a family member or partner, or was at risk of losing it); 3) no work-related household income (the woman’s household income came from public assistance, relatives, friends, or a charity); 4) absence of standard health insurance. Women with the specific health insurance provided to people who have very low income (Couverture Maladie Universelle, CMU) or illegal status (Aide Médicale d’Etat, AME) were not considered to have standard health insurance, although they were entitled to free prenatal and pregnancy care. Social deprivation was also characterized by a synthetic quantitative index adapted from the one built in the 2010 French National Perinatal Survey [21]. This deprivation index (DI) is the sum of the four indicators listed above selected by a multiple correspondence analysis, ranking women within four classes: 0) no deprivation factor; 1) one deprivation factor; 2) two deprivation factors; 3) three or four deprivation factors.

### Definition of inadequate PCU

Based on previously proposed PCU indexes, such as those by Kessner and Kotelchuck [22], we designed a new index adapted to the French standards issued by the French National Authority for Health (HAS). This index defined the inadequacy of PCU according to three key elements of prenatal care: timing of the initiation of care, the number of scheduled prenatal visits (according to the duration of the pregnancy), and the performance of scheduled ultrasound examinations. For pregnancies carried to term, HAS recommends 8 prenatal visits and 3 ultrasound examinations. PCU was considered inadequate if it began after 12 weeks of gestation, or if it included less than 50% of the number of prenatal visits expected according to duration of pregnancy, or if the first-trimester ultrasound examination or both the second- and third- trimester examinations were missing. For women who arrived in France after the beginning of the pregnancy, the GA at initiation of care, the number of scheduled prenatal visits and ultrasound examination before inclusion were considered to determine the adequacy of PCU.

### Statistical analysis

The women’s characteristics were first described globally and according to their DI category. Differences in the distribution of women’s characteristics according to their DI were tested by Student’s *t*-test for quantitative variables, and the chi-square test or Fisher’s exact test (when fewer than 5 events were expected) for categorical variables.

We then used logistic regression models and univariate and multivariate analyses to examine the associations between social deprivation and inadequate PCU. The potential confounding factors were the standard factors associated both with social deprivation and PCU (maternal age, paternal age, number of children from previous pregnancies, planned pregnancy, mode of conception), and other factors that further characterized the mother’s socioeconomic status, including education level (in four classes: none or any amount of primary schooling, completed middle school, high school, university), and immigration status, assessed with four variables: the woman’s region of birth (France, French overseas districts and territories, other European countries, North Africa, sub Saharan Africa, and other), recent immigration (i.e., arrived in France less than 12 months before pregnancy began), undocumented immigration status (including women in the process of being regularized), and the presence of a language barrier, assessed by the research assistant at enrollment (woman did not understand spoken French at all or in part or spoke French not at all or with difficulty).

The associations between maternal and paternal age with PCU was not linear. These variables were thus modeled according to their graphical distribution and then analyzed in the multivariate model as categorical covariates; categories were chosen with the likelihood ratio test.

We tested relevant interactions between social deprivation and other characteristics of the women for the risk of inadequate PCU. The interaction was statistically significant for region of birth (*P* < .003). The multivariate analyses were therefore stratified for the woman’s region of birth in three categories: France (mainland and overseas territories), North Africa, and sub-Saharan Africa. The other regions of birth (*n* = 1363, 13.9%) were not considered in the stratified multivariate analysis because the areas were too heterogeneous.

Two multivariate logistic regression models were built: in model A, social deprivation was characterized by the DI in four classes; in model B, the four binary deprivation factors were used. Models A and B were adjusted for the potential confounding factors listed above. The three variables describing migrants (recent immigration, undocumented status, and linguistic barrier) were not included in the models for the stratum “born in France”.

Goodness-of-fit of the models was assessed by the Hosmer-Lemeshow test.

### Management of missing data

No variable had more than 5% of missing data. To limit the loss of information, multiple imputations by chained equations (MICE) were used for all the variables included in the multivariate models; 30 datasets were created using those variables and birthplace.

All statistical tests were two-tailed and the threshold for statistical significance was set at a probability value of < .05. Analyses were performed with Stata v10.0 software (Stata Corporation, College Station, TX).

## Results

Among the 10,419 women included in the PreCARE cohort; 336 with multiple pregnancies and 119 with pregnancies that ended before 22 weeks of gestation were excluded. An additional 194 were subsequently excluded from the analysis: 89 women because we lacked data about their PCU or their social condition both at the beginning and at the end of the pregnancy, 104 women who were lost to follow-up, and one who withdrew her consent. A total of 9770 women were finally analyzed among the 9963 eligible for this analysis (98.1%).

Among the 9770 women in the cohort, 3419 (35%) met at least one of the four criteria for social deprivation: 2315 (23.7%) had no standard health insurance, 1612 (16.5%) had insecure housing, 1583 (16.2%) had no work-related household income, and 449 (4.6%) were isolated (Table 1).

**Table 1:** Women’s characteristics according to their social deprivation index

		Deprivation index^a^				Total
		0	1	2	3	
*N* (%)		6351 (65.0)	1773 (18.2)	913 (9.4)	728 (7.5)	9770
Mean maternal age (SD)**		31.6 (5.0)	30.0 (5.8)	29.1 (6.5)	29.3 (6.2)	30.8 (5.5)
Mean father’s age (SD)**		35.5 (6.8)	35.3 (8.0)	34.5 (8.3)	34.0 (8.4)	35.3 (7.3)
BMI >30**		11.3	15.0	14.2	17.5	12.6
Schooling Level**	≤ Primary school	3.2	10.1	13.2	18.8	6.6
	Middle school	13.5	25.4	30.9	32.6	18.6
	High school	20.8	30.6	31.8	30.6	24.3
	University	62.5	33.9	24.1	18.1	50.5
Number of children ≥ 3**		7.5	13.0	12.8	10.7	9.8
Pregnancy**	Expected	82.6	71.2	59.6	48.7	75.8
	Unexpected	16.8	27.2	37.9	44.9	22.8
	Unwanted	0.6	1.6	2.5	6.4	1.4
Mode of conception**	Spontaneous	96.1	98.1	98.0	99.5	96.9
	Ovulation induction	1.2	0.9	1.0	0.4	1.1
	ART	2.7	1.1	1.0	0.1	2.1
High-risk pregnancy^b^*		13.1	13.6	14.1	17.5	13.6
Tobacco before pregnancy*		18.4	14.8	16.4	15.6	17.3
Tobacco during pregnancy		8.6	9.2	11.2	10.1	9.0
Alcohol before pregnancy**		0.4	0.5	1.3	1.9	0.6
Alcohol during pregnancy**		2.1	1.7	2.0	5.0	2.2
Cannabis during pregnancy		0.3	0.5	0.6	0.6	0.4
HIV infection**		0.8	2.0	2.2	3.2	1.3
Maternal birth place**	France	55.9	30.2	28.5	16.2	45.7
	French overseas	2.0	1.5	2.2	1.0	1.8
	Europe (others)	4.7	5.8	4.4	5.9	5.0
	North-Africa	20.2	28.0	25.8	18.2	22.0
	Sub-Saharan Africa	9.7	20.8	28.4	50.1	16.5
	Others	7.5	13.7	10.8	8.6	9.0
Social isolation**		0.0	6.6	8.1	35.6	4.6
Poor or insecure housing condition**		0.0	24.7	54.0	93.1	16.5
Not work-related household income**		0.0	17.2	62.6	96.0	16.2
No permanent health care insurance**		0.0	51.7	75.7	96.8	23.7
Linguistic barrier**		6.6	17.8	16.9	22.4	10.8
Undocumented migrant**		0.7	12.7	18.5	43.6	7.7
Recent immigration**		3.4	9.1	11.6	26.0	6.9

All values are percentage unless specified

*SD* standard deviation, *BMI* body mass index, *DOM-TOM* French overseas departments and territories, *HIV* human immunodeficiency virus, *ART* assisted reproductive therapy

*p < 0.05 **p < 0.001

^a^Deprivation index: simple sum of 4 deprivation dimensions: Social isolation, Poor or insecure housing condition, Not work-related household income, and No permanent heath care insurance

^b^High-risk pregnancy is defined by at least one of the following item: diabetes, HIV infection, hypertension, thromboembolic event, heart disease, coagulopathy, brain aneurism, uterine malformation, Graves’ disease, autoimmune disease, alloimmunisation, nephropathy, homozygote sickle cell anemia, respiratory failure and adverse obstetrical history (pre-eclampsia, late miscarriage, cerclage, still birth, neonatal death, birth weight <2500 grams)

In addition, 752 (7.7%) were undocumented migrants, 674 (6.9%) had arrived in France less than a year before the beginning of the pregnancy, and 1055 (10.8%) had a linguistic barrier. Comparison of social, demographic, lifestyle and medical characteristics according to the DI shows significant differences for most characteristics.

PCU was classified as inadequate for 2176 women (23.3%); 6.2% had no visit before 12 weeks of gestation and 4.4% had less than half the recommended visits; 18.0% of the population had no first-trimester ultrasound examination, and 3.6% had neither a second- nor a third-trimester ultrasound examination. The percentage of women with inadequate PCU differed significantly by region of birth: 17.3% for women born in France, 26.9% for women born in North Africa, and 34.7% for women born in sub-Saharan Africa (*P* < .001).

**Fig. 1:**
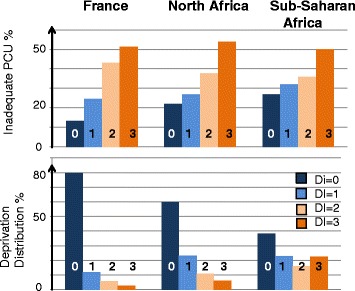
Inadequate PCU and social deprivation according to the woman’s region of birth. PCU: prenatal care utilization; DI: deprivation index: simple sum of 4 deprivation dimensions: Social isolation, Poor or insecure housing condition, Not work-related household income, and No permanent health care insurance

In the univariate analysis, each of the four social deprivation variables was significantly associated with inadequate PCU, and the relative risk (RR) for inadequate PCU showed a social gradient according to the DI (level 0: reference; level 1 RR = 1.6, 95% CI 1.5–1.8; level 2 RR = 2.3, 95% CI 2.1–2.6; level 3 RR = 3.1, 95% CI 2.8–3.4) (Table 2 and Fig. 1).

**Table 2:** Inadequate PCU according to maternal characteristics

		Inadequate PCU			
		*N*	%	Crude RR	95%CI
Total		2176/9357^a^	23,3		
Deprivation index^b^	0	1016	16.7	1	–
	1	464	27.3	1.6	1.5–1.8
	2	337	28.1	2.3	2.1–2.6
	3	359	51.8	3.1	2.8–3.4
Social isolation		201	48.8	1.8	1.6–2.1
Poor or insecure housing condition		626	41.1	2.1	1.9–2.2
Not work-related household income		651	43.0	2.2	2.1–2.4
No health care insurance		844	37.9	2.0	1.9–2.2
Maternal age (years)	<20	89	50.9	2.6	2.2–3.0
	20–25	398	32.4	1.7	1.5–1.8
	25–30	645	23.2	1.2	1.1–1.3
	30–40	910	19.6	1	–
	≥40	115	23.3	1.2	1.0–1.4
Father’s age (years)	<25	159	39.6	1.9	1.7–2.2
	25–40	1347	20.9	1	–
	≥40	550	24.2	1.2	1.1–1.3
Schooling Level	≤ Primary school	244	39.9	2.5	2.2–2.8
	Middle school	549	31.9	2.0	1.8–2.2
	High school	582	25.8	1.6	1.4–1.8
	University	745	16.2	1	–
Number of children ≥ 3		308	35.1	1.6	1.4–1.8
Pregnancy	Expected	1358	19.1	1	–
	Unexpected	739	34.9	1.8	1.7–2.0
	Unwanted	71	53.8	2.8	2.4–3.3
Spontaneous conception		2133	23.5	1	–
Ovulation induction		12	12.3	0.5	0.3–0.9
ART		24	12.9	0.5	0.4–0.8
High-risk pregnancy^c^		311	24.3	1.1	0.9–1.2
Birth place	France	725	17.1	1	–
	French overseas	38	22.6	1.3	0.9–1.8
	Europe (others)	114	24.8	1.5	1.2–1.7
	North-Africa	557	26.9	1.6	1.4–1.7
	Sub-Saharan Africa	533	34.7	2.0	1.8–2.2
	Others	196	23.2	1.4	1.2–1.6
Linguistic barrier		361	35.9	1.7	1.5–1.8
Undocumented migrant		305	41.8	1.9	1.8–2.1
Recent immigration		300	47.0	2.2	2.0–2.4

*PCU* prenatal care utilization, *ART* assisted reproductive therapy

^a^Data on PCU were missing for 413 women

^b^Cf. Table 1

^c^Cf. Table 1

After adjustment and stratification by region of birth, the DI was associated with inadequate PCU for women born in France and to a lesser extent for women born in North Africa. For women born in sub-Saharan Africa, who had higher prevalence rates of social deprivation and inadequate PCU, only DI class 3 remained significantly associated with inadequate PCU (Table 3). In the 3 stratum of birth region, inadequate PCU was associated with unplanned and unwanted pregnancy and among migrant women with recent immigration.

**Table 3:** Adjusted relative risks of inadequate PCU according to deprivation index and other maternal characteristics

Maternal region of birth		France		North Africa		Sub-Saharan Africa	
		*N* = 4628		*N* = 2140		*N* = 1606	
Inadequate PCU %		17.3%		26.9%		34.7%	
		aRR	95% CI	aRR	95% CI	aRR	95% CI
Deprivation index^a^	0	1	ref	1	ref	1	ref
	1	1.3	1.1–1.6	1.1	0.9–1.3	1.1	0.9–1.3
	2	1.9	1.6–2.3	1.4	1.1–1.7	1.1	0.9–1.4
	3	2.1	1.6–2.6	1.7	1.3–2.1	1.3	1.0–1.6
Maternal age	<20	1.4	1.2–2.3	0.7	0.3–1.7	1.2	0.9–1.7
	20–25	1.4	1.1–1.8	1.2	1.0–1.6	1.3	1.1–1.7
	25–30	1.2	1.0–1.4	1.0	0.9–1.2	1.1	0.9–1.3
	30–40	1	ref	1	ref	1	ref
	≥40	1.0	0.7–1.3	1.0	0.8–1.4	0.8	0.6–1.1
Father’s age	<25	1.3	1.0–1.5	1.1	0.7–1.8	1.1	0.8–1.6
	25–40	1	ref	1	ref	1	ref
	≥40	1.0	0.8–1.3	0.9	0.8–1.1	1.0	0.8–1.1
Schooling level	≤ Primary school	1.5	0.9–2.5	1.5	1.2–2.0	1.1	0.9–1.3
	Middle school	1.3	1.1–1.6	1.4	1.1–2.0	1.1	0.9–1.3
	High school	1.2	1.0–1.4	1.1	1.0–1.4	1.1	0.9–1.3
	University	1	ref	1	ref	1	ref
Number of children ≥ 3		1.2	1.1–1.2	1.1	1.0–1.2	1.1	1.1–1.2
Pregnancy	Expected	1	ref	1	ref	1	ref
	Unexpected	1.5	1.3–1.8	1.5	1.3–1.7	1.3	1.2–1.5
	Unwanted	1.9	1.3–2.7	1.7	1.2–2.4	1.6	1.2–2.0
Spontaneous pregnancy		1	ref	1	ref	1	ref
Ovulation induction		0.5	0.2–1.3	0.8	0.2–2.9	1.5	0.7–3.2
ART		1.0	0.6–1.8	0.7	0.3–1.6	0.9	0.4–1.9
Linguistic barrier		–	–	1.2	1.0–1.4	1.1	0.9–1.3
Undocumented migrants		–	–	1.1	0.9–1.3	1.2	0.9–1.4
Recent immigration		–	–	1.7	1.4–2.0	1.7	1.4–1.9

Multivariate Modem A using multiple imputation

Data on birth place were missing for 33 patients and data on PCU were missing for 413 women

*PCU* prenatal care utilization, *aRR* adjusted relative risk, *CI* Confidence interval, *ref* reference

^a^Deprivation index: Cf. Table 1

Associations between inadequate PCU and the different deprivation factors are detailed in Table 4. Complete cases analysis were also performed and results were not different from those presented here in which multiple imputations are used to account for missing data.

**Table 4:** Adjusted relative risks of inadequate PCU according to deprivation factors and other maternal characteristics

Maternal region of birth		France		North Africa		Sub-Saharan Africa	
		*N* = 4628		*N* = 2140		*N* = 1606	
Inadequate PCU %		17.3%		26.9%		34.7%	
		aRR	95% CI	aRR	95% CI	aRR	95% CI
Social isolation		1.0	0.7–1.4	1.0	0.7–1.3	1.0	0.8–1.2
Poor or insecure housing condition		1.3	1.1–1.5	1.0	0.8–1.2	1.2	0.9–1.4
Not work-related household income		1.3	1.1–1.6	1.3	1.1–1.5	1.2	1.0–1.4
No permanent health care insurance		1.4	1.2–1.6	1.3	1.1–1.5	0.9	0.8–1.1
Maternal age	<20	1.7	1.2–2.3	0.6	0.2–1.9	1.2	0.8–1.6
	20–25	1.4	1.1–1.8	1.2	1.0–1.6	1.3	1.1–1.6
	25–30	1.2	1.0–1.4	1.0	0.9–1.2	1.1	0.9–1.3
	30–40	1	ref	1	ref	1	ref
	≥40	1.0	0.7–1.3	1.0	0.8–1.4	0.8	0.6–1.2
Father’s age	<25	1.3	1.0–1.5	1.2	0.7–1.9	1.1	0.8–1.6
	25–40	1	ref	1	ref	1	ref
	≥40	1.0	0.8–1.3	0.9	0.8–1.1	1.0	0.8–1.1
Schooling level	≤ Primary school	1.5	0.9–2.5	1.5	1.2–2.0	1.1	0.8–1.3
	Middle school	1.3	1.1–1.6	1.4	1.1–2.0	1.1	0.9–1.3
	High school	1.2	1.0–1.4	1.1	1.0–1.4	1.1	0.9–1.3
	University	1	ref	1	ref	1	ref
Number of children ≥ 3		1.2	1.1–1.2	1.1	1.0–1.2	1.1	1.1–1.2
Pregnancy	Expected	1	ref	1	ref	1	ref
	Unexpected	1.5	1.3–1.8	1.5	1.3–1.7	1.3	1.1–1.5
	Unwanted	1.9	1.3–2.7	1.8	1.3–2.7	1.6	1.2–2.0
Spontaneous pregnancy		1	ref	1	ref	1	ref
Ovulation induction		0.5	0.2–1.3	0.8	0.2–2.9	1.5	0.7–3.2
ART		1.0	0.6–1.8	0.7	0.3–1.6	0.9	0.5–1.9
Linguistic barrier		–	–	1.2	1.0–1.5	1.1	0.9–1.3
Undocumented migrants		–	–	1.1	0.8–1.3	1.2	1.0–1.4
Recent immigration		–	–	1.6	1.3–1.9	1.7	1.4–1.9

Multivariate Model B using multiple imputation

Data on birth place were missing for 33 patients and data on PCU were missing for 413 women

*PCU* prenatal care utilization, *aRR* adjusted relative risk, *CI* Confidence interval, *ref* reference

Deprivation index: Cf. Table 1

Women were asked about the reasons they did not have more or specific appointments. Of the 5 proposed items (unawareness of pregnancy, transportation difficulties, financial reasons, lack of knowledge or understanding of availability or need for prenatal care, and lack of utility of prenatal care), the most frequently cited reason was that they had not known they were pregnant (14.1% of the women with inadequate PCU). This proportion increased with the DI (*P* < .001), reaching 19.9% in women with a DI of 3. The second most frequent reason given was that prenatal care served no purpose (6.2%); this response was not correlated to the DI (*P* = .53). Most women with inadequate PCU (67.9%) reported none of these five reasons.

## Discussion

Despite the supposedly protective policies in France, we found that social deprivation is associated with inadequate PCU here. The strength of the association and the aspects of deprivation involved varied according to women’s migration status. Inadequate PCU was also associated with recent immigration, with unplanned and unwanted pregnancy, independently of others social conditions.

In most studies, the definition of social condition is limited to only one of its multiple dimensions or is geographic/ecological rather than individual (Townsend index [23] or the Index of Multiple Deprivation [24]). A strength of this study is that the complex and multidimensional aspects of social deprivation were considered in the individual definition we used. At both enrollment and after delivery, we sought to collect several social characteristics that allowed an accurate assessment of the social condition of each woman. To identify a social gradient, we were also able to use a DI adapted from the one created from data collected in a national representative sample of pregnant women [21]. Splitting the effect of deprivation in each of its dimensions allowed us to assess the association between each of these dimensions and PCU and in particular to show no evident association between social isolation and PCU.

Another strength of this study is its large sample size and geographic setting — an area chosen to allow us to recruit numerous disadvantaged women and large samples of women born in the geographic areas most prominent in the French population. The translation of questionnaires and availability of interpreters testify to the substantial effort made to avoid loss of information from women unable to write or speak French and should have limited the potential bias due to underrepresentation of non-French speaking women. Furthermore, its prospective design and very low rate of loss to follow-up attest to the high quality of the data collected.

Our study must nonetheless be interpreted in the light of some limitations. As we chose to conduct this cohort study in an urban area marked by a high prevalence of social deprivation, where the staff of obstetrics departments are accustomed to caring for women with social problems and social agencies designed to help women, our conclusions may not be strictly the same in other settings where deprivation prevalence is lower. Regardless of the specific local context, we believe that these results should be useful in developing programs and methods to overcome barriers to adequate PCU, at least in high-resource countries.

Our assessment of social conditions was based on self-report. Women may have over- or under-reported their social difficulties, which might bias the association between barriers and inadequate prenatal care. We cannot estimate the risk or direction of this bias.

The analysis of the social data available for the women excluded from the analysis because of the lack of data about their PCU or who were lost to follow-up showed a slight increased proportion of women having a deprivation index at 2 or 3. Even though the proportion of women excluded is very low (1.9% of eligible women) and therefore unlikely to generate a selection bias. Such bias, if present, would have underestimated the strength of the association between unfavorable social conditions and inadequate PCU, any consequence that do not call into question our results.

The factors associated with inadequate PCU identified in this study varied somewhat in each of the geographical subgroups of women we considered. The issue of social deprivation in high-resource countries cannot be reduced to its immigrant populations: 20.4% of the women in this cohort who were born in France also met at least one of the four criteria for social deprivation and among them, social deprivation was highly correlated with poor PCU. Nonetheless, our results show that this issue is sharpest among immigrants.

Recent immigration was one of the factors most strongly associated with inadequate PCU in the principal subgroups of migrants studied. This finding underlines that recent immigrants are more likely than others to remain apart from the health care system, especially at the beginning of their pregnancy. The particularly higher risk of inadequate PCU for the women born in sub-Saharan Africa compared to the other geographic groups is cause for concern and might be implicated in the poorer perinatal outcomes concordantly reported in this subgroup of migrant women in several high-resource countries [25, 26]. It should be noted that the association of social deprivation with PCU was lowest in this group — but probably because the rate of inadequate PCU was already very high among the women in this group with no deprivation factors. This finding might also reflect our definition of deprivation, which selects a group with significant social vulnerabilities. Women from sub-Saharan Africa who do not meet it may nonetheless still present risk factors for inadequate PCU, such as recent migration or an unplanned or unwanted pregnancy.

Different hypotheses may help to explain this difference between women from North Africa and women from sub-Saharan Africa. First, women born in North Africa may have available to them an older, more structured, and more helpful community in France, than do women from sub-Saharan Africa. Immigration to France began from North Africa long before it did from sub-Saharan Africa. Second, as suggested by the literature [18], cultural differences in the representation of prenatal care should also be considered. Women born in Africa, especially in sub-Saharan Africa, are often reported to have poor PCU rates in European countries [27]. This is not, however, specific to African women and indeed appears to be a common characteristic among many migrant women born in low-resource countries with inadequately developed health care systems [18, 28].

Although we hypothesized that linguistic barriers might be an important obstacle to PCU, we found that poor French had a very limited association with inadequate PCU. This finding might be due to the regular presence of interpreters in the hospitals participating in the PreCARE cohort study. Beyond the availability of interpreters, cooperation among social workers, public authorities, and health professionals is necessary to improve the early participation of these women in prenatal care, regardless of their social deprivation, as defined here.

Lack of standard health insurance was associated with inadequate PCU after adjustment for confounders for women born in France and in North Africa. Other French authors have reported lack of health insurance at the beginning of pregnancy to be an important risk factor [29]. Public health efforts to provide access to prenatal care are essential and have shown good results, as illustrated by Medicaid in the US [30]. Nonetheless, despite a supposed universal access to health services in France, as in other countries of the European Union [19], as well as Brazil [31], and Canada [2], inequalities in PCU persist. The lack of awareness that there is universal access to health care, particularly among families who may not have had prior experience with health care services, could explain these results.

Women meeting our deprivation criteria had higher rates of unplanned and unwanted pregnancies than did women with a DI of 0 (Table 1) and unplanned and unwanted pregnancy were the strongest independent risk factors of inadequate PCU in French native and immigrant women (Table 3). Higher rates of unplanned pregnancy have previously been described for socially disadvantaged women [32]. Lack of knowledge of pregnancy has also been described as a major determinant of late PCU [19], especially among women with low incomes [16] or born abroad [18, 27]. Unplanned pregnancy may thus be due both to the accumulation of vulnerabilities and to cultural differences [33].

Women in situations of social deprivation and from minority groups are more likely to have high-risk pregnancies, because of their higher likelihood of medical risk factors [34, 35]. In the PreCARE cohort study, 17.5% of the women with a DI of 3 had a high-risk pregnancy because of medical risk factors, compared with 13.1% of those women with a DI of 0 (*P* < .05). Early initiation of prenatal care is thus even more important in this population than in the general population. We must therefore consider how to intensify preconception education in the general population and specifically among women not educated in France to enable women to be better prepared for pregnancy and to encourage them to recognize it early. Such education could also help them to avoid unwanted pregnancy, which is also associated with deprivation and increased risk of inadequate PCU [36].

In this study we did not consider cultural and psychological factors, a lacuna that might limit our interpretation of women’s behavior, either associated with or independent of markers of social deprivation. However, a qualitative socio-anthropological study, also part of the TRAJECTOIRES project, is underway and should enable us to round out this epidemiologic approach.

## Conclusions

These results underline the social inequality of PCU and the association between social deprivation and the risk of inadequate PCU in a country where access to health care is supposed to be universal and equal for all. Identification of the factors independently associated with inadequate PCU is an important step in the identification of barriers to PCU and in the development of measures to overcome them.

## Additional files

Additional file 1: Inclusion questionnaire. Self-administered questionnaire completed at enrolment in the cohort. (PDF 286 kb)

Additional file 2: Post partum questionnaire. Self-administered questionnaire during post partum hospitalization. (PDF 302 kb)

## Abbreviations

CI: Confidence interval
DI: Deprivation index
HAS: French national authority for health
MICE: Multiple imputations by chained equations
OECD: Organisation for economic co-operation and development
PCU: Prenatal care utilization
RR: Relative risk

## References

[1] Cantwell R, Clutton-Brock T, Cooper G, Dawson A, Drife J, Garrod D (2011). Saving mothers’ lives: reviewing maternal deaths to make motherhood safer: 2006–2008. The eighth report of the confidential enquiries into maternal deaths in the United Kingdom. BJOG.

[2] Daoud N, O’Campo P, Minh A, Urquia ML, Dzakpasu S, Heaman M (2015). Patterns of social inequalities across pregnancy and birth outcomes: a comparison of individual and neighborhood socioeconomic measures. BMC Pregnancy Childbirth.

[3] Vrijheid M, Dolk H, Stone D, Abramsky L, Alberman E, Scott JE (2000). Socioeconomic inequalities in risk of congenital anomaly. Arch Dis Child.

[4] Blumenshine P, Egerter S, Barclay CJ, Cubbin C, Braveman PA (2010). Socioeconomic disparities in adverse birth outcomes: a systematic review. Am J Prev Med.

[5] Villar J, Ba’aqeel H, Piaggio G, Lumbiganon P, Belizán JM, Farnot U (2001). WHO antenatal care randomised trial for the evaluation of a new model of routine antenatal care. Lancet.

[6] Lindmark G, Berendes H, Meirik O (1998). Antenatal care in developed countries. Paediatr Perinat Epidemiol.

[7] Blondel B, Marshall B (1998). Poor antenatal care in 20 French districts: risk factors and pregnancy outcome. J Epidemiol Community Health.

[8] Herbst MA, Mercer BM, Beazley D, Meyer N, Carr T (2003). Relationship of prenatal care and perinatal morbidity in low-birth-weight infants. Am J Obstet Gynecol.

[9] Partridge S, Balayla J, Holcroft CA, Abenhaim HA (2012). Inadequate prenatal care utilization and risks of infant mortality and poor birth outcome: a retrospective analysis of 28,729,765 U.S. Deliveries over 8 years. Am J Perinatol.

[10] Arima Y, Guthrie BL, Rhew IC, De Roos AJ (2009). The impact of the First Steps prenatal care program on birth outcomes among women receiving Medicaid in Washington State. Health Policy (New York).

[11] Ricketts SA, Murray EK, Schwalberg R (2005). Reducing low birthweight by resolving risks: results from Colorado’s prenatal plus program. Am J Public Health.

[12] Joseph JG, El-Mohandes AAE, Kiely M, El-Khorazaty MN, Gantz MG, Johnson AA (2009). Reducing psychosocial and behavioral pregnancy risk factors: results of a randomized clinical trial among high-risk pregnant African American women. Am J Public Health.

[13] Elam-Evans LD, Adams MM, Gargiullo PM, Kiely JL, Marks JS (1996). Trends in the percentage of women who received no prenatal care in the United States, 1980–1992: contributions of the demographic and risk effects. Obstet Gynecol.

[14] Gayral-Taminh M, Daubisse-Marliac L, Baron M, Maurel G, Rème J-M, Grandjean H (2005). [Social and demographic characteristics and perinatal risks for highly deprived mothers]. J Gynecol Obstet Biol Reprod (Paris).

[15] Heaman M, Bayrampour H, Kingston D, Blondel B, Gissler M, Roth C (2013). Migrant women’s utilization of prenatal care: a systematic review. Matern Child Health J.

[16] Braveman P, Marchi K, Egerter S, Pearl M, Neuhaus J (2000). Barriers to timely prenatal care among women with insurance: the importance of prepregnancy factors. Obstet Gynecol.

[17] Krans EE, Davis MM, Palladino CL (2013). Disparate patterns of prenatal care utilization stratified by medical and psychosocial risk. Matern Child Health J.

[18] Alderliesten ME, Vrijkotte TGM, van der Wal MF, Bonsel GJ (2007). Late start of antenatal care among ethnic minorities in a large cohort of pregnant women. BJOG.

[19] Delvaux T, Buekens P, Godin I, Boutsen M (2001). Barriers to prenatal care in Europe. Am J Prev Med.

[20] Steffen M (2016). Universalism, responsiveness, sustainability — regulating the French health care system. N Engl J Med.

[21] Opatowski M, Blondel B, Khoshnood B, Saurel-Cubizolles M-J (2016). New index of social deprivation during pregnancy: results from a national study in France. BMJ Open.

[22] Kotelchuck M (1994). An evaluation of the Kessner adequacy of prenatal care index and a proposed adequacy of prenatal care utilization index. Am J Public Health.

[23] Townsend P, Phillimore P, Beattie A (1988). Health and deprivation: inequality and the North.

[24] Jordan H, Roderick P, Martin D (2004). The index of multiple deprivation 2000 and accessibility effects on health. J Epidemiol Community Health.

[25] Urquia ML, Glazier RH, Gagnon AJ, Mortensen LH, Nybo Andersen A-M, Janevic T (2014). Disparities in pre-eclampsia and eclampsia among immigrant women giving birth in six industrialised countries. BJOG.

[26] Urquia ML, Glazier RH, Blondel B, Zeitlin J, Gissler M, Macfarlane A (2010). International migration and adverse birth outcomes: role of ethnicity, region of origin and destination. J Epidemiol Community Health.

[27] Cresswell JA, Yu G, Hatherall B, Morris J, Jamal F, Harden A (2013). Predictors of the timing of initiation of antenatal care in an ethnically diverse urban cohort in the UK. BMC Pregnancy Childbirth.

[28] Mda Santiago CF, Figueiredo MH (2015). Immigrant women’s perspective on prenatal and postpartum care: systematic review. J Immigr Minor Health.

[29] Blondel B, Dutilh P, Delour M, Uzan S (1993). Poor antenatal care and pregnancy outcome. Eur J Obs Gynecol Reprod Biol.

[30] Fullerton J, Bader J, Nelson C, Shannon R (2004). Prenatal care in the Paso del Norte border region. J Perinatol.

[31] Bernardes ACF, da Silva RA, Coimbra LC, Alves MTSS de B, Queiroz RC de S, Batista RFL (2014). Inadequate prenatal care utilization and associated factors in São Luís, Brazil. BMC Pregnancy Childbirth.

[32] Hulsey TM (2001). Association between early prenatal care and mother’s intention of and desire for the pregnancy. J Obstet Gynecol Neonatal Nurs.

[33] Meikle SF, Orleans M, Leff M, Shain R, Gibbs RS (1995). Women’s reasons for not seeking prenatal care: racial and ethnic factors. Birth.

[34] Zeitlin J, Combier E, Levaillant M, Lasbeur L, Pilkington H, Charreire H (2011). Neighbourhood socio-economic characteristics and the risk of preterm birth for migrant and non-migrant women: a study in a French district. Paediatr Perinat Epidemiol.

[35] Makgoba M, Savvidou MD, Steer PJ (2012). An analysis of the interrelationship between maternal age, body mass index and racial origin in the development of gestational diabetes mellitus. BJOG An Int J Obstet Gynaecol.

[36] Wilcox LS, Koonin LM, Adams MM (1999). Quality measures for unintended pregnancy prevention in health care services: opportunities and challenges. Womens Health Issues.

