# Supplementary material for: Association between maternal social deprivation and prenatal care utilization: the PreCARE cohort study
Source: BMC Pregnancy Childbirth. 2017 May 16;17:126. doi: 10.1186/s12884-017-1310-z (PMC5433136; doi:10.1186/s12884-017-1310-z)
Supplement: Supplementary file 1 — Inclusion questionnaire. Self-administered questionnaire completed at enrolment in the cohort. (PDF 286 kb) [file 12884_2017_1310_MOESM1_ESM.pdf]

Dear Mrs, Ms,

Your participation in completing this questionnaire is extremely important; it allows us to gain a better understanding of the influence of various factors (such as your family living situation or your social welfare cover etc.) on the development of your pregnancy and the health of your new-born child.

The information that you provide us will be analysed in a completely anonymous manner; neither your doctor nor the patient care team will be given access to this questionnaire.

If you have any additional questions, please do hesitate to contact the scientific study manager, Dr Azria on 01.40.25.76.69.

Your participation is on a voluntary basis and you may refuse to complete this questionnaire.

Once again, thank you for devoting your time to this research.

The PreCARE cohort research team

Cohort of pregnant women
QUESTIONNAIRE: Start of pregnancy

1-What was your family living situation at the start of pregnancy (when you became aware that you were pregnant)?

☐ Living as a couple:
☐ Married ☐ Civil partnership ☐ Cohabiting

☐ Not living as a couple:
☐ Divorced ☐ Separated ☐ Widow ☐ Single

☐ Other, please specify...

2-Was there anyone you could rely on, (at the start of your pregnancy) to assist you with the birth?

☐ No, no-one
☐ Husband, partner
☐ Friend/family member
☐ Community support
☐ Other, please specify...

3-What type of accommodation were you living in at the start of your pregnancy?
☐ Stable, not short-term (no risk of losing your accommodation in the short-term)
☐ Short-term or instable: if yes, please specify.
☐ Friend/family member
☐ Hotel (personally financed)
☐ Squat
☐ Homeless
☐ Institutional accommodation (emergency outreach service for the homeless, hostel, maternity home, association)
☐ Other, please specify..……………………………………………………………………

4-What is the post code of the town where you were living at the start of your pregnancy:
|__|__|__|__|__|
***Or the* Name of the town………………………………………………….**

5-What were your main sources of income (yourself and your partner) at the start of your pregnancy? (several answers possible)
☐ Work
☐ Family/friends
☐ Social benefits
☐ None
☐ Other, please specify..………………………………………………………………………………

6-What social welfare cover were you entitled to at the start of your pregnancy?
☐ Social security
☐ Social security + mutual insurance
☐ CMU (health care coverage for people on low incomes)
☐ AME (state medical assistance)
☐ Life-threatening emergency assistance
☐ Other, please specify..………………………………………………………………………………

7- How many children do you have? |__|__|

Have any of these children been taken into social care? ☐ Yes ☐ No

If yes, how many: |__|__|

8- When you found out that you were pregnant, how many weeks (or months) pregnant were you?

|__|__| **weeks** ***or*** |__| months

9- What is your current nationality?

☐ French
☐ Nationality of a European Union member country
☐ Another nationality

10- If you are not French or a member of the European Union, what was your situation in relation to the French or European authorities (at the start of your pregnancy)?

(Strictly confidential data governed by professional secrecy)

☐ Permanent residence permit
☐ Temporary residence permit or a receipt for a temporary residence permit
☐ Provisional residence permit
☐ A valid short or long term tourist visa
☐ Awaiting regularisation (request in progress)
☐ No residence permit request
☐ Other, please specify..

11- What is your place of birth?

☐ Metropolitan France
☐ Overseas department and territory
☐ Europe
☐ North Africa
☐ Sub-Saharan or Black Africa
☐ Middle East or Near East
☐ Asia
☐ Other, please specify...

12- What is your origin? (several answers possible)
☐ Metropolitan France
☐ Overseas department and territory
☐ Europe
☐ North Africa
☐ Sub-Saharan or Black Africa
☐ Middle East or Near East
☐ Asia
☐ Other, please specify...

12a- ***If you are of African origin:***
Have you travelled to Sub-Saharan Africa (Black Africa) over the last 4 years?
☐ Yes ☐ No
if yes, please specify
Countries visited: ....
Start date of your trip: |__|__| /|__|__|/ |__|__|__|__|

Duration of the trip: ……………………………………….

12a- *If you are of another origin:*

Have you travelled to Sub-Saharan Africa (Black Africa) over the last 6 months?

☐ Yes ☐ No

If yes, please specify

Countries visited: …………………………………………….

Start date of your trip: |__|__| /|__|__|/ |__|__|__|__|

Duration of the trip: ……………………………………….

13- Do you live in France? ☐ Yes ☐ No

If yes

☐Since my birth

Or since: |__|__| /|__|__|/ |__|__|__|__|

14 – What is the place of birth of the father of your future child?

☐ Metropolitan France
☐ Overseas department and territory
☐ Europe
☐ North Africa
☐ Sub-Saharan African
☐ Asia Minor
☐ Asia
☐ Don't know
☐ Other, please specify..…………………………………………………………………………

15 – What is the year of birth of the father of your future child? |__|__|__|__| (year)

Or the age of the father of your future child: |__|__|

☐ Don't know

16-What level of education did you attain?

☐ Never received schooling
☐ Primary school
☐ Secondary school (11-16 years of age)/GCSE or equivalent
☐ Sixth form college (general and technical education) (from lower to upper-sixth form)
☐ Higher education (following A-levels or equivalent)

***Or* the Number of years of education (since beginning primary school)** |__|__|

17-Is this a pregnancy that you: (several answers possible)

☐ Wanted
☐ Didn't want
☐ Was unplanned

18-With whom did you have initial medical contact in France for the monitoring of your pregnancy: doctor, midwife?

☐ General practitioner
☐ Out-patient obstetrician-gynaecologist or midwife (health practice, MCW, health centre etc.)
☐ Maternity-unit obstetrician-gynaecologist or midwife (hospital), excluding emergency consultations
☐ Emergency consultation
☐ Sonologist

☐ Other, please specify: ....

19- What was the date of your first medical appointment during your pregnancy?

|__|__| /|__|__|/ |__|__|__|__|

20-On what date did you register for the delivery of your child in a maternity unit?

|__|__| /|__|__|/ |__|__|__|__|

21- Consumption of Alcohol

a) Since the start of your pregnancy, how often do you consume alcohol?

☐ Never
☐ Once per month or less
☐ 2 to 4 times per month
☐ 2 to 3 times per week
☐ At least 4 times per week

b) Since the start of your pregnancy, how many glasses of alcohol do you consume on a typical day that you drink alcohol?

☐ Less than 3
☐ 3 or 4
☐ 5 or 6
☐ 7 to 9
☐ 10 or more

c) Since the start of your pregnancy, how often do you drink six glasses or more of alcohol on a special occasion?

☐ Never
☐ Less than once per month
☐ Once per month
☐ Once per week
☐ Every day or most days

Thank you for taking the time to complete this questionnaire
