# Supplementary material for: Association between maternal social deprivation and prenatal care utilization: the PreCARE cohort study
Source: BMC Pregnancy Childbirth. 2017 May 16;17:126. doi: 10.1186/s12884-017-1310-z (PMC5433136; doi:10.1186/s12884-017-1310-z)

Dear Mrs, Ms….,

Your participation in completing this questionnaire is extremely important; it allows us to gain a better understanding of the influence of various factors (such as your family living situation or your social welfare cover etc.) on the development of your pregnancy and the health of your new-born child.

You may have already completed this type of questionnaire at the start of your pregnancy.
It is however very important for us to find out if there has been any change in your situation and to understand the methods of monitoring your pregnancy.

The information that you provide us will be analysed in a completely anonymous manner; neither your doctor nor the patient care team will be given access to this questionnaire.

If you have any additional questions, please do hesitate to contact the scientific study manager, Dr Azria on 01.40.25.76.69.

Your participation is on a voluntary basis and you may refuse to complete this questionnaire.
Once again, thank you for devoting your time to this research.

The PreCARE cohort research team

# Cohort of pregnant women
## QUESTIONNAIRE: End of pregnancy

**1- Which method of monitoring did you receive during your pregnancy?**
☐ Monitored at the hospital on a doctor's recommendation although out-patient monitoring was intended (Doctor, midwife, MCW)
☐ Registered at the hospital with out-patient monitoring then hospital monitoring
☐ Registered and monitored at the hospital
☐ Not monitored
☐ Solely emergency consultation

**2-Which maternity unit did you register with for giving birth?**
☐The maternity unit where you gave birth?
☐Another maternity unit, which one:……………………………………..
☐None

**3- Did you change maternity units during the course of your pregnancy?**
☐ Yes ☐ No
*if yes, why?*

☐ Maternal medical reason
☐ Foetal medical reason
☐ Maternal care preference
☐ Moving home
☐ A financial problem
☐ Other, please specify: .................

**4- How many out-patient consultations did you receive?**
(Doctor, midwife, MCW)
- overall I__I__I
- including I__I__I prior to your registration with the maternity unit

**5- How many hospital consultations did you receive?**
(excluding emergency consultations, excluding functional testing): I__I__I

**6-How many emergency consultations did you receive during your pregnancy?**
- overall I__I__I
- including I__I__I prior to your initial registration with the maternity unit

**7- How many different doctors or midwives have you seen during your pregnancy?**
**(in the maternity unit or as an out-patient, excluding emergency consultations)** I__I__I

**8- How many consultations did you receive during the month prior to giving birth**?
(excluding pre-anesthesia and ultrasound scan consultations)

Emergency consultations n= |__|
Functional testing n= |__|
Start date of functional tests |__|__| /|__|__|/ |__|__|__|__|
Other consultations n= |__|

**9- How many ultrasound scans did you have during your pregnancy?**
Overall |__|__|
Including |__| Emergency ultrasound scans

**10- Did you have:**

**- a scheduled consultation (non emergency) prior to 3 months pregnancy** ☐YES ☐NO

**- at least 3 scheduled consultations during your pregnancy** **(excluding ultrasound scan and emergency consultations)** ☐YES ☐NO

**- always a period of below one month and a half between 2 scheduled consultations** **(excluding ultrasound scan and emergency consultations)** ☐YES ☐NO

***If you answered "no" to one of the 3 options above,*** this is due to:
☐ Lack of knowledge or understanding of the recommended monitoring
☐ Financial reasons
☐ Consultations considered not to be necessary
☐ Travel problems (transport, childcare etc.)
☐ No knowledge of pregnancy
☐ Other, please specify: ............................

**11-What was your family living situation the month prior to giving birth**?

☐ Living as a couple:
☐Married ☐ Civil partnership ☐ Cohabiting

☐ Not living as a couple:
☐Divorced ☐ Separated ☐ Widow ☐ Single

☐ Other, please specify.........................................................................................

**12-Was there anyone you could rely on to assist you with the birth?**
☐ No, nobody
☐ Husband or partner
☐ Friend/family member
☐ Community support
☐ Other, please specify.........................................................................................

**13-What type of accommodation were you living in the month prior to giving birth?**
☐ Stable, not short-term (no risk of losing your accommodation in the short-term)
☐ Short-term or instable: if yes, please specify.
- ☐ Friend/family member
- ☐ Hotel (personally financed)
- ☐ Squat
- ☐ Homeless
- ☐ Institutional accommodation (emergency outreach service for the homeless, hostel, maternity home, association)
- ☐ Other, please specify...........................................................................................

**14-What social welfare cover were you entitled to at the time of giving birth?**
☐ Social security
☐ Social security + mutual insurance
☐ CMU (health care coverage for people on low incomes)
☐ AME (state medical assistance)
☐ Life-threatening emergency assistance
☐ Other, please specify.......................................................................................................

**15- If you are not French or a member of the European Union, what was your situation in relation to the French or European authorities at the time of giving birth?**
(Strictly confidential data governed by professional secrecy)
☐ Permanent residence permit
☐ Temporary residence permit or a receipt for a temporary residence permit
☐ Provisional residence permit
☐ A valid short or long term tourist visa
☐ Awaiting regularisation (request in progress)
☐ No residence permit request
☐ Other, please specify.......................................................................................................

**16-What were your main sources of income (yourself and your partner) at the time of giving birth?**
(**several answers possible)**
☐ Work
☐ Family/friends
☐ Social benefits
☐ None
☐ Other, please specify.......................................................................................................

**17-Did you have a social worker during your pregnancy?** ☐ **YES** ☐**NO**

**18-Did you have a psychologist during your pregnancy?** ☐ **YES** ☐**NO**

**19-Have you participated in any other research in the medical field?** ☐ **YES** ☐**NO**

If yes, what is the name of this research:..........................................

**Thank you for taking the time to complete this questionnaire**

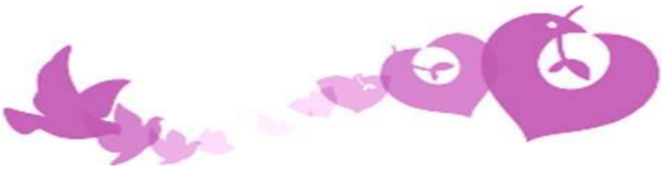

Supplement: Supplementary file 2 — Post partum questionnaire. Self-administered questionnaire during post partum hospitalization. (PDF 302 kb) [file 12884_2017_1310_MOESM2_ESM.pdf]
